# Synchronous infiltrating ductal carcinoma and primary extramedullary plasmacytoma of the breast

**DOI:** 10.1186/1477-7819-7-43

**Published:** 2009-04-24

**Authors:** Shui Cao, Hong-Gang Kang, Yan-Xue Liu, Xiu-Bao Ren

**Affiliations:** 1 Department of Biotherapy, Tianjin Cancer Hospital & Institute, Tianjin Medical University, Tianjin, PR China; 2 Department of Pathology, Tianjin Cancer Hospital & Institute, Tianjin Medical University, Tianjin, PR China

## Abstract

**Background:**

Extramedullary plasmacytomas are seldom solitary and usually progress to diffuse myelomatosis. Plasmacytomas of the breast are rare, especially when not associated multiple myeloma. Synchronous infiltrating ductal carcinoma and primary extramedullary plasmacytoma of the breast have not previously reported.

**Case presentation:**

A 27-years-old woman with an untreated upper outer quadrant breast mass for 1-year was referred to our cancer hospital for surgical evaluation of increasing breast pain. Postoperatively, microscopic examination revealed an infiltrating ductal carcinoma complicated by an extramedullary plasmacytoma divided by fibrous tissue in one section. Following surgery, the patient received chemotherapy for the carcinoma and radiotherapy for the plasmacytoma.

**Conclusion:**

In this case, careful histopathology examination was essential to make the correct diagnosis and therapy for these synchronous lesions. The patient finished chemotherapy and radiotherapy without significant adverse effects.

## Background

Extramedullary plasmacytoma is described most frequently in the upper respiratory tract but it may also be found in the oral cavity, gastrointestinal tract, lung, lymph nodes, skin, and subcutaneous tissue [1,2]. Involvement of the breast is rare. While infiltrating ductal breast cancer is very common throughout the world, synchronous primary extramedullary plasmacytoma and breast cancer have not previously been reported.

## Case presentation

A 27-years-old woman with an untreated upper outer quadrant breast mass for 1-year was referred to our cancer hospital for surgical evaluation of increasing breast pain. She had no history of bone pain, weight loss, fatigue, fever or other systemic complaints, and no family history of breast cancer. On physical examination there were no skin changes or nipple discharges, and the mass was firm, freely moveable, and nontender. Mammography confirmed a well-defined 5.2 cm mass in upper outer quadrant of the right breast. There were no satellite lesions. Laboratory tests including complete blood count, total protein, glucose, hepatic and renal function panels were normal. Because primary breast carcinoma was suspected patient agreed to a modified radical mastectomy. There was no extension from the capsulated masses to pectoral muscles or chest wall, and no axillary lymph node involvement.

**Figure 1:**
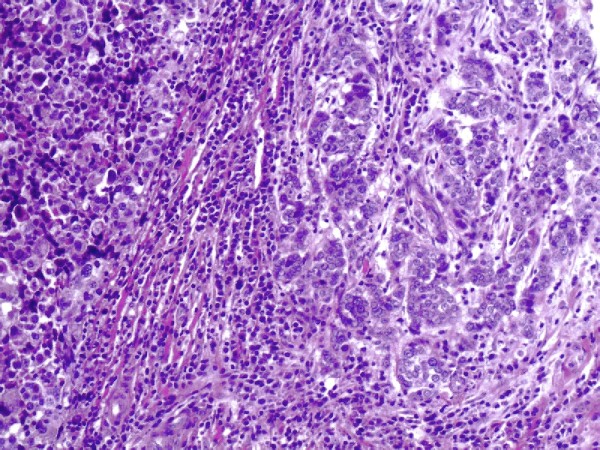
**Extramedullary plasmacytoma and breast cancer were divided by fibrous tissue**. (Hematoxylin & Eosin×40).

**Figure 2:**
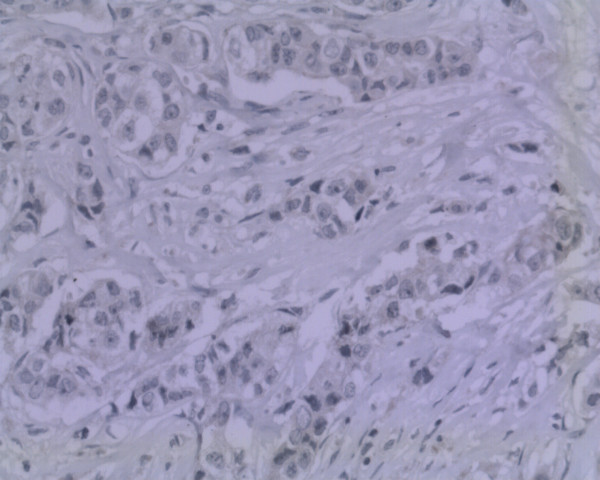
**Immunohistochemical stain for Her-2 was negative ×40**.

**Figure 3:**
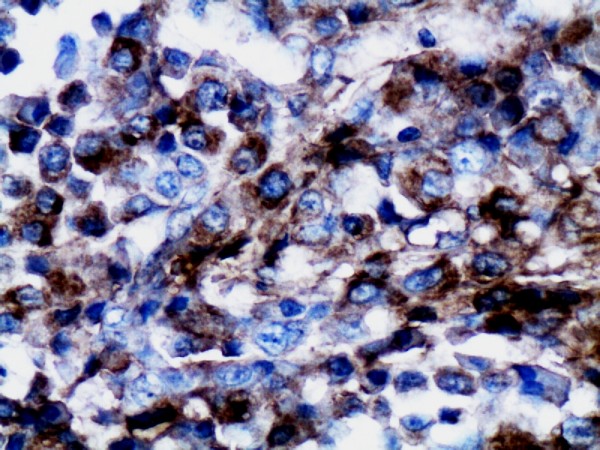
**Plasma cells are diffusely and strongly positive for light kappa chains ×200**.

Gross pathology examination revealed a soft, pale, encapsulated gritty mass surrounded by normal breast tissue, measuring 5.0 cm × 4.0 cm × 2.5 cm. Histopathological examination showed an infiltrating ductal carcinoma complicated by an extramedullary plasmacytoma divided by fibrous tissue in one section (Figure 1). Immunohistochemical stains were negative for estrogen, P170, and progesterone receptors. Her-2 was negative (Figure 2). Nuclear prognostic marker (Ki-67) showed 25% to 50% nuclear expression. Topoisomerase-IIα (+<5%), CylinD-1, Cytokeratin, and S-100 were negative. The tumor cells were strongly positive for light kappa chains (Figure 3), and negative for light lambda, delta, and my chains.

After the surgery and pathologic findings, serum immunoglobulins were measured and found to be: IgG 10.03 g/l (8.0–16 g/l), IgA 1.34 g/l (0.7–3.3 g/l), IgM 1.44 g/l (0.5–2.2 g/l). No Bence Jones or other M components were detected in the urine. Serum calcium and phosphorus were normal. Posterior iliac crest bone marrow biopsy was negative for plasma cells. PET and CT scans, except for absence of the right breast, did not detect other lesions.

The patient was offered and accepted chemotherapy and radiotherapy based on previous research results about infiltrating ductal carcinoma and case reports about extramedullary plasmacytoma.

## Discussion

Primary soft tissue extrameullary plasmacytoma (SEP) is uncommon and is defined as a malignant tumor of plasma cells arising in the soft tissue in the absence of bone involvement. It can occur in any organ as a solitary form of plasma cell neoplasm. [1] Some authors consider SEP to be unrelated to multiple myeloma. Although SEP can arise throughout the body, almost 80% to 90% of the cases arise in the head and neck areas. [2]. Approximately 70% occur in patients with multiple myeloma. Since Visalia reported the first case in 1928 [3], approximately sixty-three cases have been described in published literature [1-13]. More than two-thirds of the lesions were unilateral in breast [2,4]. Extramedullary plasmacytomas are seldom solitary and usually progress to diffuse myelomatosis as a first manifestation of multiple myeloma [7,14,15], or recurrence of multiple myeloma [4]. Plasmacytomas of the breast are rare, especially those not associated with multiple myeloma.

The circulating paraproteins vary in type and include kappa and lambda light-chain patterns, IgA and IgG [4]. In this patient paraproteins were absent in serum and urine, but strongly positive for kappa light chains in the lesion.

In our case the tumor was not more than 5 cm in greatest dimension, and tests for estrogen receptor, progesterone receptor, and Her-2 were negative. As recommended for primary breast cancer in "Practice Guidelines in Oncology – 2008", post surgery the patient received chemotherapy. In this case it was three cycles of AC→T (doxorubicin/cyclophosphamide followed by paclitaxel) and no hormonal therapy. It is generally accepted that plasmacytomas are radiosensitive, and excellent long-term results have been reported with local control rates following radiotherapy of 79% to 90%, and 10-years survival rates from 50% to 100% [15-17]. Mendenhall reported a threshold dose of 40 Gy for local control [10]. Local recurrence develops most frequently in the first five years of follow-up but maybe found many years later [8]. Based on these previous reports, after chemotherapy this patient received a dose of 50 Gy radiation therapy.

This case emphasizes the importance of excision biopsy and immunohistochemical panel in the differential diagnosis and correct therapy of breast masses. The infiltrating ductal carcinoma and extramedullary plasmacytoma were divided by fibrous tissue so it is possible they were two independent diseases, juxtaposed by accident in one clinical lesion. As this is the first case reported, a causal relationship between synchronous extramedullary plasmacytoma and infiltrating ductal carcinoma of the breast must remain speculative.

## Consent

Patient's permission was obtained for publishing her case records.

## Competing interests

The authors declare that they have no competing interests.

## Authors' contributions

SC was involved in treatment of the patient, collected case details, literature search and prepared the article. HGK was involved in treatment of the patient, collected case details, literature search and helped in preparation of manuscript. YXL was involved in pathological diagnosis and figures, wrote the pathological part of the manuscript. XBR was involved in treatment planning of the patient and manuscript preparation. All authors read and approved the final manuscript.
